# Main indications of clozapine in clinical practice: A literature review

**DOI:** 10.1192/j.eurpsy.2025.2257

**Published:** 2025-08-26

**Authors:** P. Rivero Rodriguez, M. Martinez Grimal, J. J. Tascón Cervera, A. M. Morales Rivero, F. A. Rodriguez Batista

**Affiliations:** 1 Psychiatry, Hospital Universitario de Gran Canaria Doctor Negrin, Las Palmas de Gran Canaria, Spain

## Abstract

**Introduction:**

Clozapine is an atypical and complex antipsychotic that appears to benefit from actions on multiple neurotransmitter systems. While its mechanisms of action are not fully understood, this broad spectrum of activity accounts for clozapine’s superior efficacy in treating refractory schizophrenia and other conditions.

**Objectives:**

The aim of this paper is to review the main indications of clozapine and its applications in clinical practice, as well as to highlight key considerations for its safe and effective management.

**Methods:**

A systematic review of the scientific and clinical literature on clozapine was conducted. The review included databases such as PubMed and Cochrane, covering articles from the past 20 years. The scientific evidence obtained was analyzed and synthesized.

**Results:**

Findings indicate that clozapine remains the treatment of choice for patients with treatment-resistant schizophrenia, showing a superior response rate compared to other antipsychotics. Additionally, its effectiveness in reducing suicidal behaviors in patients with schizophrenia and related disorders has been identified. The indications also extend to psychosis in Parkinson’s disease, substance use disorders, and a wide range of psychiatric and neurological disorders.

**Conclusions:**

Clozapine is essential in the treatment of refractory schizophrenia and in reducing suicide risk. Its broad mechanism of action, affecting multiple neurotransmitters, allows its use in secondary psychotic disorders and complex comorbidities, such as Parkinson’s disease. However, its use is associated with significant risks, necessitating rigorous monitoring of adverse effects.

**Disclosure of Interest:**

None Declared

